# Promotion of Cardiovascular Homeostasis by the Perivascular Adipose Tissue Secretome

**DOI:** 10.3390/ijms262010173

**Published:** 2025-10-20

**Authors:** Olivia R. Whittaker, Matthew D. Lynes, Ilka Pinz, Lucy Liaw

**Affiliations:** MaineHealth Institute for Research, Scarborough, ME 04074, USA; olivia.whittaker@tufts.edu (O.R.W.); matthew.lynes@mainehealth.org (M.D.L.); ilka.pinz@mainehealth.org (I.P.)

**Keywords:** cardiovascular, extracellular vesicles, secretome, signaling, cardiometabolic

## Abstract

Perivascular adipose tissue (PVAT) is a unique fat depot that is distributed around blood vessels, contiguous with the vascular adventitia. Due to this proximity, it serves as a local source of adipokines and vasoregulatory factors. Similar to other adipose depots, PVAT is responsive to changes in metabolic state and, at least in mice, can transition to a thermogenic adipocyte phenotype depending on metabolic health. Cardiovascular disease risk is highly correlated with metabolic health and increases substantially in individuals with obesity or metabolic syndrome. Cardiovascular diseases, including atherosclerosis/coronary artery disease, aortic aneurysm, hypertension, arterial stiffening, and heart failure, have been associated with PVAT dysregulation. Understanding the cardiovascular protective effects of healthy PVAT can provide ways to modify disease progression to re-establish functional homeostasis. This review focuses on experimental studies that specifically define a signaling axis between PVAT and the cardiovascular system that provide cardioprotection. Our focus is primarily on the secreted contents of extracellular vesicles that initiate this adipose signaling axis and regulation of extracellular vesicle release by the trafficking molecule, RAB27a. We review the current literature on human and mouse studies and major categories of PVAT-derived signaling components including microRNAs, lipids, and proteins that contribute to cardiovascular homeostasis.

## 1. Introduction

Cardiovascular disease (CVD) is a heterogeneous group of pathologies involving the heart and vascular system, that collectively represent the leading cause of death in the United States and increasing globally. Conditions ranked by level of mortality include coronary artery disease (~40%), stroke (~18%), other CVD (~17%), hypertensive disease (~14%), heart failure (~9%), and diseases of arteries (~3%). Behaviors and medical conditions such as smoking, poor diet, inactivity, excessive alcohol consumption, high blood pressure, high cholesterol, and obesity all increase the risk for CVD [1]. Communication from the vascular microenvironment to the cardiovascular system is a key mediator of tissue homeostasis. Perivascular adipose tissue (PVAT) has been identified as a source of paracrine signaling factors that either protect the cardiovascular system or participate in pathologic cellular dysregulation, increasing the risk for CVD. PVAT surrounds blood vessels and is an important regulator of vascular tone by secreting cytokines which interact with the blood vessel wall [2]. In conditions of cardiometabolic dysfunction and obesity, PVAT secretes pro-inflammatory cytokines, such as leptin, MCP-1, and TNF-alpha, that lead to proatherogenic states, concepts which have been previously reviewed [3,4]. While there is extensive literature associating disease-related adipose tissue phenotypes, this review will focus on PVAT-derived factors that are considered cardioprotective, which we define as having effects that suppress cellular changes associated with pathology. We highlight PVAT-derived factors where evidence suggests they may be secreted through signaling microvesicles released extracellularly.

The secretome of a cell or tissue is the combined array of bioactive factors released extracellularly to induce autocrine or paracrine signaling [5]. The cellular secretome is regulated by metabolic state and is fundamental in the body’s ability to maintain homeostasis by facilitating communication between major organ systems and tissues. These mediators of communication can be proteins, peptides, RNA species such as mRNAs, microRNAs and lncRNAs, lipids, hormones, and metabolites. The secretome can be further categorized into free circulating factors or those packaged within extracellular vesicles. These vesicles are characterized by size and biogenesis: exosomes (ranging from 30 nm to 100 nm) are formed within the endosomal network and are released through the plasma membrane [6]; micro vesicles/microparticles (ranging from 200 nm to 1 μm) are directly shed from the plasma membrane; and lastly, apoptotic bodies (ranging from 1 μm to 4 μm) are released as membrane blebs.

There are multiple proteins and processes involved in the formation and secretion of exosomes from cells. Intracellular vesicles form larger multivesicular bodies that fuse with the plasma membrane and result in the release of intraluminal vesicles as exosomes. Sorting of cargo within these vesicles is complex and not well characterized; known plasma membrane microdomain proteins, lipids, and structures involved include caveolae, clathrin, ceramide, lipid rafts, dynamin, neutral sphingomyelinase, small GTPases (including RAB family members), and many other proteins related to the cytoskeleton and plasma membrane [7]. Formation of intraluminal vesicles within the multivesicular bodies occurs via at least two pathways—endosomal sorting complex required for transport (ESCRT)-dependent or -independent. The ESCRT pathway contributes to the transport of multivesicular bodies and sorting of exosomal cargo [8]. Interestingly, ceramide contributes to the ESCRT pathway and ESCRT-independent intraluminal vesicle budding [9], which also involve another RAB family member, RAB31 [10].

Exosomes and extracellular vesicles have been reviewed for their roles in cardiovascular diseases as biomarkers and potential therapeutic targets [11,12,13]. Likewise, adipose tissue-derived exosomes and their target tissues and organs have been widely defined and reviewed in the recent literature [14,15,16,17]. We recently identified a novel connection between a member of the Rab GTPase family, RAB27A, and cardiac and vascular dysfunction in vivo [18]. Despite the primary role of RAB27A in exosome biogenesis and secretion and its requirement for metabolic homeostasis, very little is known about RAB27A function in adipose tissue and its signaling functions. However, RAB27A is an interesting target as a mediator of adipose–cardiovascular signaling, as described below.

## 2. RAB27A Function in Exosome Regulation and Cardiovascular Health

The release of exosomes through membrane docking and fusion relies on protein signal cascades. Rab GTPases, particularly RAB27A and RAB27B, play essential roles in protein trafficking, mediating multivesicular endosome fusion with the plasma membrane, and exosome release into the extracellular space [19,20,21]. In melanoma cells, loss of RAB27a also changes the protein content of secreted extracellular vesicles or promotes the section of a molecularly distinct sub-class of exosomes [22]. Human missense mutations in *RAB27A* cause Griscelli syndrome type 2 [23,24,25,26], and affected individuals have variable levels and onset [27] of impaired melanocyte trafficking, leading to albinism, silver/gray hair coloring, and impaired immune cell degranulation, often leading to neurological damage [26]. Lifespan for individuals with Griscelli syndrome type 2 is short, with improved prognosis after hematopoietic stem cell transplantation. Because many patients do not survive childhood, age-associated conditions such as heart or vascular disease have not been associated with this population. However, using human genetic data from the Common Metabolic Diseases Knowledge Portal, genetic variants of human *RAB27A* were found to be associated with body mass index, hypertension, and myocardial infarction [18]. These data suggest that in humans, RAB27A function contributes to cardiometabolic homeostasis [28].

RAB27A is produced in human and mouse PVAT [18], and we found that suppression of RAB27A by siRNA in human preadipocytes inhibited adipocyte differentiation [29]. While *Rab27a* generally has a wide expression pattern throughout the body, in the mouse it is not expressed in the heart, while in blood vessels, it is expressed in PVAT, vascular smooth muscle cells, and endothelial cells [18]. The importance of RAB27A in exosome release has been established in loss of several *Rab27a* mouse strains. In one *Rab27a*-null mutant mouse strain, bone marrow-derived endothelial progenitor cells lacking RAB27A had decreased viability, proliferation, and reduced exosome release [30,31]. The cells lacking RAB27A did not induce therapeutic improvement when transplanted after myocardial infarction, unlike bone marrow-derived cells from wild type mice [20].

In a different, independently targeted *Rab27a*-null strain, global loss of RAB27A was associated with age-related cardiovascular defects [18]. At eight weeks of age, *Rab27a*-null male mice had increased vasoconstriction and reduced vasodilation. By 20 weeks of age, the males exhibited a cardiomyopathy phenotype. These loss-of-function data show that RAB27A has cardio- and vasculo-protective effects. RAB27A loss in male mice was associated with delayed glucose clearance in a glucose tolerance test, suggesting changes in insulin regulation or signaling [32,33]. It is possible that the absence of RAB27A may alter the secretome, leading to downstream changes in the phenotype of targeted cells. The loss-of-function phenotypes could be a primary effect of production within the heart and blood vessels themselves or metabolic changes in other tissues that then regulate cardiovascular function. As PVAT-derived molecules have been associated with protection against CVD, we hypothesize that RAB27A plays a role in the release of these cardioprotective factors.

While RAB27a is a candidate regulator of extracellular vesicle signaling in the PVAT–to–heart/vascular axis, it is important to note that this hypothesis needs further in vivo testing. The components of the secretome or extracellular vesicles that are described below have functional activity, and some have been validated as exosomal cargo. However, exact mechanisms and regulation of these RNA, lipid, and proteins specifically by RAB27a-mediated cellular release mechanisms in PVAT are currently under investigation. While we developed a conditional, floxed RAB27a mouse strain [18], currently, there are no PVAT-specific Cre strains that can distinguish RAB27a function in PVAT from other adipose tissues. Ongoing studies are focused on the approach of ex vivo characterization of the secretome of human PVAT.

## 3. Cardioprotective RNAs Secreted from PVAT

RNAs released in extracellular vesicles, including microRNAS (miR), long non-coding RNAs, mitochondrial RNAs, and mRNAs, play key roles in communication between PVAT and target cells. This communication impacts physiological and pathological vascular development as well as homeostasis.

While PVAT-derived RNA species associated with the progression of cardiovascular pathologies have been identified, new studies have begun to identify RNA contents attenuating and protecting against disease (Table 1). A study by Sun et al. [34] aimed to identify how miRNAs from adipose endothelial cells regulate gene expression and modulate the development and progression of cardiometabolic phenotypes. miRNA-181b expression in endothelial cells in adipose tissue of obese mice was significantly reduced after 12 weeks on a high-fat diet, and obese mice treated with miRNA-181b showed improved glucose tolerance and insulin sensitivity. Further, the administration of miRNA-181b attenuated endothelial dysfunction, enhanced the production of eNOS, and exerted anti-inflammatory effects [34]. Another study by Liu et al. analyzed the effects of PVAT-derived extracellular vesicles on vascular homeostasis and showed that miR-382-5p was expressed at lower levels in PVAT-derived vesicles from patients with coronary atherosclerotic heart disease than in healthy subjects. miR-382-5p reduced macrophage foam cell formation and increased the expression of cholesterol efflux transporters ABCA1 and ABCG1 downstream of PPARγ, consistent with a protective effect of this microRNA [35].

Administration of microRNAs through neutrophil membrane-engineered extracellular vesicles (NVEVs) provide therapeutic benefits. Wei et al. engineered NVEVs through the fusion of PVAT-derived extracellular vesicles with neutrophil membrane nanovesicles, resulting in superior targeting to regions of inflamed vasculature. miR-206-3p in NVEVs promoted regression of atherosclerotic plaques through ABCA1 cholesterol efflux and favorable vascular remodeling. Furthermore, administration of the NVEVs in rat models of atherosclerosis significantly attenuated inflammation, stabilized vascular architecture, and reduced macrophage infiltration [36]. The identification and study of protective PVAT-derived microRNAs provide potential therapeutic targets to re-establish cardiovascular homeostasis.

Long non-coding RNAs are another emerging area of interest, although few studies exist on non-coding RNAs specifically in the relationship between PVAT and the cardiovasculature. A study by Xie et al. identified promising findings on the expression of LINC01180, an immune-related lncRNA, as a potential protective factor against the development of atherosclerosis in patients [37]. In this study, LINC01180 was among eight other long non-coding RNA with immune-related functions that were elevated in PVAT from individuals with coronary heart disease. Expression of LINC01180 was found to be associated with the instability of atherosclerotic plaques, suggesting that this is a promising target. Despite the minimal research presently available, long non-coding RNAs are another promising avenue for future RNA studies that may provide important insights into the development and protection against disease.

**Table 1:** PVAT-derived cardioprotective RNA species.

RNA	Effects	Reference
miRNA-181b	Exerts anti-inflammatory effects; enhances production of endothelial NO synthase (eNOS); attenuates endothelial dysfunction	[33]
miR-382-5p	Reduces macrophage foam cell formation by mediating upregulation of cholesterol efflux transporters, ABCA1, and ABCg1	[34]
miR-206-3p	Enhances cholesterol efflux via miR-206-3p-ABCA1-dependent signaling and upregulation of cholesterol efflux transporters, ABCA1, and ABCg1	[35]
LINC01180	Protective factor against the progression of atherosclerosis	[37]

## 4. Cardioprotective Lipids Derived from PVAT

The field of lipidomics is fairly young, and there were only limited reports characterizing the lipid content of extracellular vesicles in healthy and diseased states at the time this review was written (Table 2). Studies to date have focused on the links between lipidomic profiles and the development of diseases such as cancer, specifically prostate and ovarian malignancies, and cardiometabolic diseases [38]. Membrane lipids are an integral component of exosomal vesicles generated intracellularly by the budding and fission of multivesicular endosomes [39]. Extracellular vesicles are enclosed by a lipid bilayer with a specific lipid composition of cholesterol, sphingolipids, and phospholipids; however, the extent of this specific enrichment can vary by cell type and metabolic state [39]. Extracellular vesicles thus contribute to carrying lipid cargo between cells, tissues, and organs.

### 4.1. Sphingolipids

Similar to their cells of origin, exosome membranes are asymmetrical, with the outer leaflet enriched in sphingomyelin and the inner leaflet higher in phosphatidylserine species; however, changes in cell membrane lipid organization can influence the distribution of these lipids in the exosomes they secrete [51]. Exosomes are particularly enriched in sphingolipids due to the necessity of forming a membrane with tight curvature, which requires small, conic-shaped lipids. Sphingolipids also function as lipid rafts, essential platforms that support the function of membrane-bound proteins and provide a sorting mechanism for vesicle flow from the Golgi apparatus to the plasma membrane for release [52,53]. One well-designed study by Mahmoud et al. [54] compared extracellular vesicles from healthy, lean individuals to obese individuals and found that those from healthy individuals were characterized by higher levels of sphingomyelin and phospholipids and lower levels of ceramide and free fatty acids. Previous work has already suggested that sphingomyelin and ceramides are markers of cardiovascular disease, and studies have shown a link between circulating ceramides and tissue damage in cardiometabolic diseases [55,56,57]. Circulating subclasses of sphingolipids, including sphingomyelin, ceramides, and sphingosine-1-phosphate, have been directly linked to the concurrence of diabetic cardiomyopathy, dilated cardiomyopathy, myocardial ischemic heart disease, hypertension, and atherogenesis [49,58].

While most available literature focuses on the link between circulating lipids and the progression of disease, new evidence has emerged on their role in maintaining cardiovascular homeostasis. Sphingosine-1-phosphate can protect the heart against ischemia and reperfusion injury when released together with adenosine [59,60,61]. Evidence suggests that vesicular sphingolipids and their metabolic enzymes, such as sphingomyelinases and ceramidases, contribute to extracellular vesicle signaling and action by influencing recipient cell sphingolipid levels, potentially contributing to the development of these diseases. The finding that extracellular vesicles contain enzymes that metabolize lipids into molecules with different biological activity [62] expands their metabolic significance. In addition to sphingomyelinases and ceramidases, exosomes contain phospholipase enzymes and cyclooxygenases, allowing cells to compartmentalize the production of bioactive fatty acids [63].

Exosomes are also enriched in glycosylated sphingolipids such as hexosylceramides, which can be associated with pathology [64,65,66]. In the heart, hexosylceramides are protective against the deleterious effects of ceramide accumulation [67] and they change the biophysical properties of membranes by increasing fluidity.

### 4.2. Palmitic Acid Methyl Ester

A study by Lee et al. identified the role of PVAT-derived palmitic acid methyl ester (PAME) on vascular tone, an important mediator for the progression of vascular disease [68]. Their study found that PAME, a PVAT-derived relaxing factor, induced vessel vasorelaxation via the opening of K+ channels in vascular smooth muscle cells. In addition, they discovered that a reduction in the release of PAME alongside an increase in angiotensin II in PVAT was associated with hypertension, suggesting PAME as a cardiovascular protective lipid [68].

### 4.3. Phosopholipids

The cardioprotective effects of phospholipids containing polyunsaturated fatty acids vs. monounsaturated or saturated fatty acids have been well established. Recent reviews of this topic provide an in-depth review of the cardioprotective effects of these lipids, including the anti-inflammatory properties of specific omega-3 and omega-6 polyunsaturated fatty acids [69,70]. However, more work is needed to clarify the role of polyunsaturated and monounsaturated fatty acid-containing lipids in exosomes and how exosomes may contribute to the distribution of cardioprotective lipids.

### 4.4. Plasmalogens

Several reports have shown that exosomes contain plasmalogens [71,72,73], which are ether lipids important for membrane fusion [74] and have strong anti-oxidant and anti-inflammatory functions [75,76]. The content of plasmalogens in exosomes of various origins is estimated to be up to 50% of all lipids. The plasmalogen content of exosomes released from PVAT is unknown and requires further study. However, considering the important biological roles of plasmalogens, we anticipate that they will prove to be an important aspect of cardioprotection in the PVAT–cardiovascular axis.

## 5. Protective PVAT-Derived Protein Secretome

Several secreted proteins are released from PVAT-derived cells; these are summarized in Table 3.

### 5.1. Adiponectin

Adiponectin is highly secreted from adipose tissue. It can be secreted in exosomes and also regulates exosome secretion. PVAT-derived adiponectin is an important mediator in the promotion of vascular health and the maintenance of homeostatic conditions in the cardiovascular system [96,97]. PVAT adiponectin acts as an anti-atherogenic factor by reducing vascular O2-, a free radical that can cause vascular damage, and increasing PI3/Akt-mediated eNOS phosphorylation, which increases the production of vasorelaxing factors [80]. It also attenuates the secretion of inflammatory factors through AMP-activated protein kinase inhibition of the NF-kB signaling pathway, leading to decreased cardiac inflammation and atherosclerosis. In an aging model of cardiovascular deletion of the endonuclease ERCC1, vasodilation was decreased due to decreased NO production and loss of endothelium-derived hyperpolarization. PVAT under these conditions was shown to partially compensate for decreased vasorelaxation through its secretion of adiponectin [96]. In lean human models, PVAT secretes adiponectin as a mean of protecting against the development of hypertension and diabetes by reducing peripheral vascular resistance and improving nutrient uptake into tissues [98,99]. PVAT adiponectin plays many cardiovascular protective roles by maintaining homeostasis, decreasing cardiac inflammation, inhibiting cardiomyocyte hypertrophy, regulating fibrosis, and suppressing the development of atherosclerosis [100].

The release of adiponectin is mediated by fatty acid-binding protein 4 (FABP4), a nuclear ligand importer, and its targeted receptor, peroxisome proliferator-activated factor (PPAR-γ) [101,102]. Activation of the PPAR- γ-signaling cascade in PVAT has been shown to upregulate the adiponectin gene in response to vascular oxidative stress to protect against injury and inflammation [103]. Interestingly, the literature describing the expression of PPAR- γ and FABP4 in PVAT from mice fed high-fat or high-cholesterol diets reports some discrepancies. In a study by Chatterjee et al., mice fed a high-fat diet for two weeks expressed lower levels of adiponectin, PPAR-γ, and FABP4 as compared to chow-fed mice [77]. However, Irie et al. found that mice fed a high-cholesterol diet had upregulated PPAR-γ and FABP4 expression in PVAT at the end of 12 weeks when compared to chow-fed mice [84]. It is possible that two weeks of a high-fat diet was not long enough to induce adipocyte differentiation, as FABP4 and PPAR-γ are important regulators of differentiation and adipocyte phenotype determination.

The expression of adiponectin has been reported to change based on metabolic status. Multiple studies have found an association between obesity and metabolic syndromes, and reduced levels of PVAT-derived adiponectin in comparison to non-obese individuals. Greif et al. investigated the relationship between pericardial fat volumes and CVD risk factors such as serum adiponectin, inflammatory biomarkers, and the morphology and quantity of coronary atherosclerosis. Elevated pericardial adipose tissue volumes, such as in obesity, were associated with low adiponectin levels, low high-density lipoprotein, elevated TNF-alpha levels, and increased atherosclerotic lesions [104]. Decreased levels of periadventitial adipose tissue has been associated with disease progression. High-fat, high-sucrose feeding in mice was associated with inflammatory changes and decreased adiponectin expression in periadventitial adipose tissue. These inflammatory changes and decreased adiponectin levels were associated with enhanced neointima formation after endovascular injury. Adiponectin-deficient mice showed increased neointima formation in injured blood vessels [105].

### 5.2. Endothelial Nitric Oxide Synthase

Endothelial nitric oxide synthase (eNOS) in PVAT regulates the production of nitric oxide (NO), a key vasoprotective element. Uncoupled eNOS can no longer produce NO, but instead creates superoxide, a reactive oxygen species (ROS) that causes tissue damage and inflammation. Reduced bioavailability of NO can lead to endothelial dysfunction associated with atherogenesis. This pathological mechanism has been shown in obesity, where uncoupled eNOS in PVAT results in excess ROS by NAD(P)H oxidase activation, leading to a decrease in NO production [85,87]. A study by Gil-Ortega et al. measured the expression of eNOS and NO bioavailability in mesenteric PVAT of mice fed with a high-fat diet and found undetectable expression. Conversely, superoxide levels were increased in the PVAT of obese mice, and mesenteric endothelial-dependent relaxation was significantly impaired in the high-fat diet group compared to the control [106]. Thus, PVAT eNOS plays a critical role in maintaining cardiovascular health by protecting against harmful ROS. However, the mechanism of how eNOS expression in PVAT is regulated is not fully understood and requires additional research.

### 5.3. Omentin-1

Omentin-1 is encoded by the gene *intelectin-1 (ITLN1)*, and its expression in PVAT protects against cardiovascular disease by targeting the immune system to reduce pro-inflammatory mediators [107]. Omentin-1 is an antifibrotic adipocytokine that inhibits transforming growth factor (TGF)-β, a key pro-inflammatory activator of cardiac fibroblasts in epicardial adipose tissue [89]. It decreases the levels of pro-inflammatory and pro-oxidant C-reactive protein and nitrotyrosine in PVAT and exerts endothelial beneficial effects by restoring NO levels and inhibiting oxidative stress in animal models of type 2 diabetes [88]. The cardiovascular protective effects of omentin-1 have further been modeled by its administration in disease states. Hiramatsu et al. demonstrated a reduction in lipid droplets, macrophage accumulation, atherosclerotic lesion formation, and mRNA expression of pro-inflammatory mediators, tumor necrosis factor-alpha, IL-6, and monocyte chemotactic protein-1, after omentin-1 administration in mice [107]. Furthermore, omentin-1 restored the anti-contractile action of PVAT that was lost in diabetic mice [88].

Consistent with its cardioprotective effects, some studies have found decreased omentin-1 levels in cardiovascular disease. Chen et al. observed that in patients with atrial fibrillation, omentin-1 was downregulated in epicardial adipose tissue and the right atrial appendages, while TGF-b1 was upregulated [89]. In patients with coronary artery disease, omentin-1 levels in circulation and epicardial adipose tissue were decreased and was exaggerated in fat tissue directly next to coronary stenotic segments [90]. Thus, local levels of omentin-1 are not always inversely correlated with disease state. Another example of this is the report that omentin-1 expression was increased in the epicardial adipose tissue of non-obese patients with coronary artery disease [91]. It is likely that the overall metabolic health of individuals, as well as local tissue pathology, contributes to omentin-1 production at different sites within the cardiovasculature.

### 5.4. Fibroblast Growth Factor-21

Fibroblast growth factor-21 (FGF-21), an anti-inflammatory adipokine, is secreted in large amounts by the liver and adipose tissue, but is expressed in lower amounts in the aorta and PVAT [92]. There is limited information on the role of FGF21 from PVAT on vascular homeostasis. A study by Mestres-Arenas et al. aimed to characterize whether expression of bioactive factors known to be produced in brown adipose tissue were secreted in PVAT surrounding the thoracic or abdominal aorta. In the mouse, PVAT surrounding the thoracic aorta resembles brown adipose tissue, whereas PVAT close to the abdominal aorta has a mixture of white adipose tissue and fewer brown-like adipocytes. Cytokines typically produced in brown adipose tissue were higher in the thoracic region and lower in the abdominal region [92], corresponding to their cellular morphology.

Circulating FGF21 has anti-atherosclerotic effects; it reduces lipid and cholesterol profiles, inhibits macrophage migration, inhibits foam cell formation, alleviates oxidative stress by ROS, and reduces the expression of inflammatory cytokines [108,109,110]. Furthermore, FGF21 promotes the secretion of adiponectin, which results in the reduction in endothelial dysfunction and inhibition of smooth muscle cell proliferation and foam cell formation. As a whole, these effects come together to contribute to FGF21 resistance to the progression of CVD [111,112]. FGF21 is a potential target that, when released from PVAT, could contribute to PVAT’s cardioprotective effects.

## 6. Discussion, Limitations, and Future Directions

PVAT is now understood to mediate a variety of effects on cardiovascular health and disease. There are several outstanding issues related to leveraging this knowledge to promote vascular health in human populations. Firstly, most of the work studying the molecular features in PVAT has occurred in rodent models, which are tractable for the study of metabolic disease, cardiovascular disease and injury, and aging. It is not always straightforward to translate genetic and signaling pathways from rodent models to human disease, so validation is warranted. Molecular analysis of human adipose tissues and comparisons to mouse adipose tissues have been driven through single-cell transcriptomics studies, in particular, for white [113] and beige/brown adipose tissues [114]. More recently, we have analyzed available data from human PVAT from unique cohorts of patients with cardiovascular disease, in comparison to mouse PVAT [115,116]. While there are certainly conserved phenotypes, especially in functionally mature adipocytes, we noted species diversity, especially in adipose progenitor populations. At the morphological level, human PVAT appear similar to subcutaneous white adipose tissue, although adipocyte size has been reported to be smaller. Human PVAT also expresses thermogenic markers such as FGF21, UCP-1, and PGC-1a in vivo [117], with a similar profile maintained when progenitor cells from human PVAT were derived and differentiated in vitro, compared to progenitor cells from human subcutaneous white adipose tissue [118].

**Figure 1:**
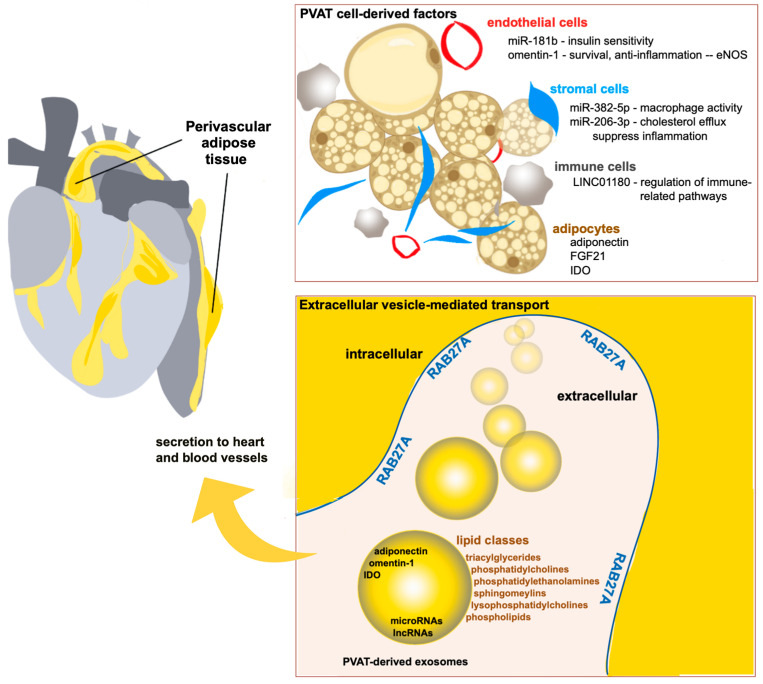
Model of PVAT-derived extracellular vesicle cardioprotective factors. PVAT is contiguous with the vascular adventitia, providing an anatomically linked mechanism for paracrine regulation of vascular smooth muscle cells of blood vessels and the heart. Multiple cell types contribute to the secretome of PVAT. For those molecules that are associated with extracellular vesicles, RAB27a is a critical regulator of multivesicular body fusion with the cellular plasma membrane, allowing for vesicular release. Several lipid classes implicated in cardioprotection have been reported to be associated with extracellular vesicles, as have adiponectin, omentin-1, and IDO.

The overall landscape of secreted paracrine factors from PVAT, especially from human tissue, remains an area of active study. Distinct from soluble paracrine factors, the packaging of diverse molecular cargo in extracellular vesicles is expected to provide a longer half-life and potentially allow cargo to be targeted to different organs [119]. There are many categories and types of secreted microvesicles [120], and often it is unclear whether specific molecules are packaged as vesicular cargo or have an association with vesicles. The ExoCarta small extracellular vesicle protein, RNA, and lipid database [41] provides a curated list of molecules experimentally associated with extracellular particles. Using this resource, we have summarized the PVAT-derived cardioprotective factors discussed as evidence of their localization in secreted extracellular vesicles (Figure 1). Of note, there is experimental evidence that some components of extracellular vesicles are conserved between rodents and humans. Adiponectin is an example of this, as it has been characterized in extracellular vesicles from adipocytes in rats [121] and from human bone marrow mesenchymal cells [122]. However, other components of extracellular vesicles have only been studied in one cell type or species and may not be broadly generalizable. There is also expected to be some cell-type specificity in cargo release within vesicles.

We propose that RAB27a production in the cardiovascular microenvironment, including in PVAT, is one molecular mechanism by which paracrine signaling occurs from adipose tissue to the heart and blood vessels via extracellular vesicle trafficking [94,95]. Many factors, whether within exosomes or secreted in soluble form, may comprise this adipose–cardiovascular signaling.

Limitations of our current knowledge include the fact that most PVAT studies have been performed in rodent models. Genetic targeting with exosome labeling is being characterized in mouse models [123,124,125,126], although it remains a challenge to target PVAT selectively and distinguish it from other adipose depots. Several key studies using human PVAT and PVAT-derived cells have been described, and continuing research in these areas are required to discover whether PVAT-derived molecules identified in model organisms are generalizable to human PVAT signaling capacity.

The role of PVAT-secreted cardioprotective molecules is a novel area of study that provides a potential future target for the treatment of CVD. While PVAT proteins have been heavily researched, there is a gap in knowledge of PVAT-derived RNAs and lipids. Further investigation into RNA and lipid exosomal contents may allow the development of a therapeutic platform for the use of endothelial cells to deliver these anti-inflammatory mediators [35]. Currently, there are emerging therapies, such as canakinumab and colchicine, that target cardiovascular inflammatory pathways and have shown promise in preclinical and clinical trials. However, these treatments are not target-specific, leading to systemic immunosuppression. This drawback can be eliminated using extracellular vesicle technology, which recent studies have begun to demonstrate successfully [35].

Like all adipose depots, PVAT alters its molecular and cellular phenotype with metabolic imbalance, which is reversible. The predicted thermogenic capacity of human PVAT, based on levels of thermogenic markers, decreases with progressive weight gain [117]. Thus, it is feasible that a variety of health interventions intended to promote healthy inflammatory and metabolic profiles will impact the PVAT secretome. One example includes the positive impact of exercise [127,128]. Indeed, one of the PVAT-derived targets of interest, FGF21, may be regulated by aerobic and endurance exercise [129,130]. It is also interesting to consider that protective effects of exercise could also be mediated by changes in the cardiovascular system that sensitize heart or vasculature to PVAT-derived signaling factors [131].

Lastly, future research is necessary to determine how intracellular exosome release proteins modify the type of content released into extracellular space, especially in human cells and tissues. RAB27a is a promising target, since it has already been identified to protect against disease in mice and is associated with human disease-causing mutations. However, it is unknown how the expression of RAB27a modifies the contents of cellular secretions under conditions of metabolic stress. More detailed molecular study of human PVAT in broader clinical populations will allow for the continued development of strategies to promote cardiovascular health and decrease the risk of cardiovascular diseases.

## 7. Methods

### 7.1. Eligibility Criteria

The inclusion criteria utilized for the article search are as follows: I. Subjects: mice and human models; II. Exposure: proteins, RNA, or lipids derived from PVAT (mice and human models); III. Outcome: protection against disease progression or upregulation in healthy physiologic states; IV. Study type: case–control; V. Study question: what are the PVAT-derived proteins, RNAs, or lipids that serve as a protective mediator against the progression of cardiovascular disease?

The exclusion criteria are as follows: I. Molecules not derived from any type of PVAT; II. PVAT-derived molecules identified with the progression of CVD or were upregulated during CVD states.

### 7.2. Search Strategy

**Figure 2:**
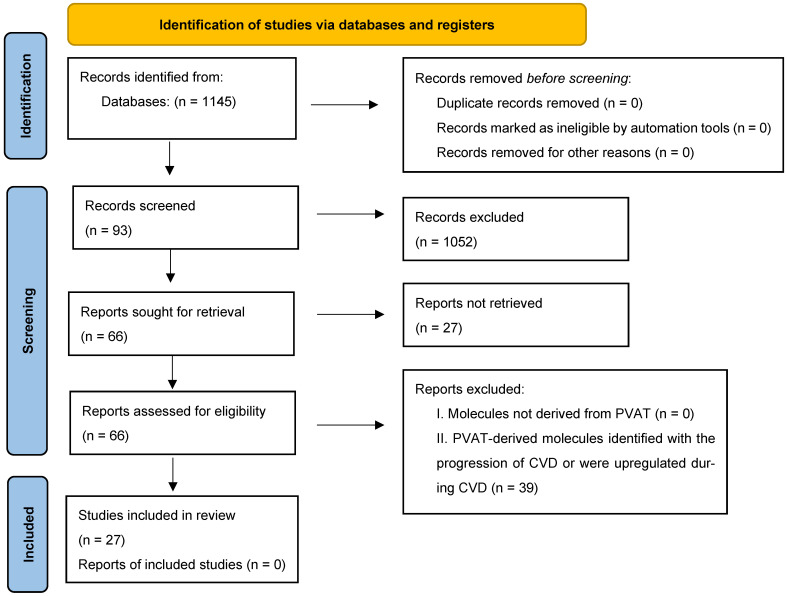
PRISMA flow diagram.

The search strategy is summarized in Figure 2. A articles were gathered through searches within Pubmed, Scopus, and Google Scholar. An advanced search strategy was utilized for PubMed, utilizing MeSH terms. Separate searches were conducted for the three molecule types.

For the search of PVAT-derived proteins in PubMed, the search terms were as follows: ((PVAT) OR ((perivascular) AND (adipose) AND (tissue))) AND ((protein) OR (proteins) OR (extracellular)) AND ((cardiovascular) OR (CVD) OR (atherosclerosis) OR (cardiometabolic)).

For the search of PVAT-derived RNA, the search terms were as follows: ((PVAT) OR ((perivascular) AND (adipose) AND (tissue))) AND ((RNA) OR (mRNA) OR (miRNA) OR (EV)).

For PVAT-derived lipids, the search terms were as follows: ((PVAT) OR ((perivascular) AND (adipose)) AND ((lipids) OR (sphingolipids) OR (phospholipids) OR (lipid)) AND ((secretion) OR (circulating) OR (blood) OR (plasma) OR (extracellular) OR (exosome) OR (exosomal)).

Searches in Scopus and Google Scholar yielded a much broader range of results compared to the PubMed search. The same search parameters for PubMed were utilized in both Scopus and Google Scholar search engines to identify relevant articles that were not included in the PubMed results. Searches were conducted up to July 2025, and references from relevant studies and review articles were included to ensure all relevant information was included.

### 7.3. Selecting Studies and Data Extraction

Article search results matching the eligibility criteria were pooled into a master Microsoft Excel spreadsheet, which highlighted the publication year, author, title, summary of key findings, background information, methods, biomarkers, and DOI. Multiple tabs were created to sort articles into their identified biomarker: proteins, RNAs, and lipid secretions. Additional articles providing background information on relevant study results were also included to help support and expand upon the importance of study conclusions.

## Figures and Tables

**Table 2:** Lipid species with evidence for cardiovascular protective activities and/or exosome localization of the major lipid class [40,41].

Lipid Class	Example Species	Cardioprotective Action	References
Triacylglycerides (TAGs) containing polyunsaturated fatty acids	TAG(18:1/18:1/22:6),	Anti-inflammatory PUFA supply; improve endothelial NO signaling	[42]
Prostaglandins containing polyunsaturated fatty acids	Prostaglandin E2-EP4	Protection from ischemic events	[43]
Phosphatidylcholines (PC) and phosphatidylethanolamines (PE) with n3-PUFA chains	PC(22:6-n3) PE(22:6-n3)	Anti-oxidant capacity and protection from ischemic events, linked with better vascular/metabolic outcomes	[44]
Plasmalogens (ether-linked Pls)	PlsPE(16:0/22:6), PlsPC(18:0/20:5)	Anti-oxidant; protect endothelial NO	[42]
Sphingosine-1-phosphate (S1P)	S1P(d18:1)	Enhances NO production, endothelial barrier, and survival signaling	[45,46]
Hexosylceramides (HexCer)	HexCer(d18:1/16:0), HexCer(d18:1/24:0)	Inverse association with CVD risk in some cohorts	[47]
Unsaturated lysophosphatidylcholines (Lyso-PCs)	Lyso-PC(18:2), Lyso-PC(20:4)	Some species linked with better vascular function	[48]
Sphingomyelins (SM, long-chain unsaturated)	SM(d18:1/24:1)	Some species inversely associated with atherosclerosis	[49,50]

**Table 3:** PVAT-derived cardioprotective secreted proteins.

Protein	Effects	Species	References
Adiponectin	An anti-inflammatory adipokine.	Human	[77]
	Protects against vascular neointima lesion formation.	Mice	[78]
	Decreased adiponectin induced inflammatory pathophysiological conditions.	Mice	[79]
	Reversed molecular interactions associated with CAD, such as the adhesion of THP-1 cells to endothelial cells and reduced expression of intercellular adhesion molecules.	Human	[80]
	Reduced expression in obese PVAT; absence contributes to NO inhibition in obesity.	Human	[81]
	An anti-inflammatory adipokine; potentially elicits beneficial effects in the pathogenesis of CAD	Human	[82]
	Contributes to myocyte hyperpolarization; releases NO to induce vasorelaxation	Mice	[83]
	Reduced pro-inflammatory cytokines like TNF-α and IL-6; acts as an anti-inflammatory, and anti-atherogenic.	Mice	[84]
Endothelial Nitric Oxide Synthase (eNOS)	Produces NO that is anti-atherogenic, by controlling vascular smooth muscle proliferation, inhibiting platelet aggregation, leucocyte adhesion, and vascular inflammation.	Mice and Humans	[85,86]
	Produces NO; Uncoupling of eNOS increases ROS production, leading to oxidative stress and inflammation; Obese/metabolic syndrome mice had higher eNOS uncoupling.	Mice	[79]
	Uncoupling contributes to generation of superoxide and impairs tonic NO release; Obese tissue has decreased NO.	Human	[81]
	Uncoupling of eNOS diminishes superoxide production; Uncoupling is a function of arginase induction and l-arginine deficiency; Diet-induced obesity leads to l-arginine and NO deficiency, and eNOS uncoupling.	Mice	[87]
Omentin-1	Recovers anti-contractile action; Improves pro-inflammatory and pro-oxidant PVAT phenotype; Restores NO and inhibits oxidative stress.	Rat	[88]
	Downregulated in atrial fibrillation; Inhibit TGF-beta1-induced cardiac fibroblast activation; Antifibrotic adipocytokine.	Human	[89]
	Decreased in patients with coronary artery disease; Decreased in fat next to coronary stenotic segments.	Human	[90]
	Increased expression in epicardial adipose in patients with coronary artery disease; plays cardioprotective role.	Human	[91]
	Increased expression in response to cold (brown fat). Mild cold-induced PVAT activation attenuates age-dependent and obesity-induced endothelial dysfunction.	Mice	[92,93]
	Caused a relaxation response in vessels.	Mice	[94]
Indoleamine 2,3-dioxygenase metabolite (IDO)	Enzymes primarily in brown fat surrounding the thoracic aorta; Depresses aortic contractility.	Rats	[95]

## Data Availability

No new data were created or analyzed in this study. Data sharing is not applicable to this article.

## Abbreviations

The following abbreviations are used in this manuscript:

CVD	cardiovascular disease
eNOS	endothelial nitric oxide synthase
ESCRT	endosomal sorting complex required for transport
FABP4	fatty acid-binding protein 4
FGF21	fibroblast growth factor 21
ITLN1	intelectin-1
NVEV	neutrophil membrane-engineered extracellular vesicles
PAME	palmitic acid methyl ester
PPAR-γ	peroxisome proliferator-activated factor
PVAT	perivascular adipose tissue
TGF	transforming growth factor

## References

[1] Martin S.S., Aday A.W., Allen N.B., Almarzooq Z.I., Anderson C.A.M., Arora P., Avery C.L., Baker-Smith C.M., Bansal N., Beaton A.Z. (2025). 2025 Heart Disease and Stroke Statistics: A Report of US and Global Data from the American Heart Association. Circulation.

[2] Antoniades C., Tousoulis D., Vavlukis M., Fleming I., Duncker D.J., Eringa E., Manfrini O., Antonopoulos A.S., Oikonomou E., Padró T. (2023). Perivascular adipose tissue as a source of therapeutic targets and clinical biomarkers. Eur. Heart J..

[3] Nosalski R., Guzik T.J. (2017). Perivascular adipose tissue inflammation in vascular disease. Br. J. Pharmacol..

[4] Kim H.W., Shi H., Winkler M.A., Lee R., Weintraub N.L. (2020). Perivascular Adipose Tissue and Vascular Perturbation/Atherosclerosis. Arterioscler. Thromb. Vasc. Biol..

[5] Zullo J., Matsumoto K., Xavier S., Ratliff B., Goligorsky M.S. (2015). The cell secretome, a mediator of cell-to-cell communication. Prostaglandins Other Lipid Mediat..

[6] Kalluri R., LeBleu V.S. (2020). The biology, function, and biomedical applications of exosomes. Science.

[7] Arya S.B., Collie S.P., Parent C.A. (2024). The ins-and-outs of exosome biogenesis, secretion, and internalization. Trends Cell Biol..

[8] Liese S., Wenzel E.M., Kjos I., Rojas Molina R., Schultz S.W., Brech A., Stenmark H., Raiborg C., Carlson A. (2020). Protein crowding mediates membrane remodeling in upstream ESCRT-induced formation of intraluminal vesicles. Proc. Natl. Acad. Sci. USA.

[9] Trajkovic K., Hsu C., Chiantia S., Rajendran L., Wenzel D., Wieland F., Schwille P., Brügger B., Simons M. (2008). Ceramide triggers budding of exosome vesicles into multivesicular endosomes. Science.

[10] Wei D., Zhan W., Gao Y., Huang L., Gong R., Wang W., Zhang R., Wu Y., Gao S., Kang T. (2021). RAB31 marks and controls an ESCRT-independent exosome pathway. Cell Res..

[11] Zhang Z., Zou Y., Song C., Cao K., Cai K., Chen S., Wu Y., Geng D., Sun G., Zhang N. (2024). Advances in the study of exosomes in cardiovascular diseases. J. Adv. Res..

[12] Guo D., Xu Y., Ding J., Dong J., Jia N., Li Y., Zhang M. (2020). Roles and Clinical Applications of Exosomes in Cardiovascular Disease. BioMed Res. Int..

[13] Reiss A.B., Ahmed S., Johnson M., Saeedullah U., De Leon J. (2023). Exosomes in Cardiovascular Disease: From Mechanism to Therapeutic Target. Metabolites.

[14] Cai M., Zhao D., Han X., Han S., Zhang W., Zang Z., Gai C., Rong R., Gao T. (2023). The role of perivascular adipose tissue-secreted adipocytokines in cardiovascular disease. Front. Immunol..

[15] Le Lay S., Scherer P.E. (2025). Exploring adipose tissue-derived extracellular vesicles in inter-organ crosstalk: Implications for metabolic regulation and adipose tissue function. Cell Rep..

[16] Wang Y., Li Q., Zhou S., Tan P. (2024). Contents of exosomes derived from adipose tissue and their regulation on inflammation, tumors, and diabetes. Front. Endocrinol..

[17] Zhao R., Zhao T., He Z., Cai R., Pang W. (2021). Composition, isolation, identification and function of adipose tissue-derived exosomes. Adipocyte.

[18] Soucy A., Potts C., Kaija A., Harrington A., McGilvrey M., Sutphin G.L., Korstanje R., Tero B., Seeker J., Pinz I. (2024). Effects of a Global Rab27a Null Mutation on Murine PVAT and Cardiovascular Function. Arterioscler. Thromb. Vasc. Biol..

[19] Ostrowski M., Carmo N.B., Krumeich S., Fanget I., Raposo G., Savina A., Moita C.F., Schauer K., Hume A.N., Freitas R.P. (2010). Rab27a and Rab27b control different steps of the exosome secretion pathway. Nat. Cell Biol..

[20] Zhou W., Zheng X., Cheng C., Guo G., Zhong Y., Liu W., Liu K., Chen Y., Liu S., Liu S. (2021). Rab27a deletion impairs the therapeutic potential of endothelial progenitor cells for myocardial infarction. Mol. Cell. Biochem..

[21] Gurung S., Perocheau D., Touramanidou L., Baruteau J. (2021). The exosome journey: From biogenesis to uptake and intracellular signalling. Cell Commun. Signal..

[22] Guo D., Lui G.Y.L., Lai S.L., Wilmott J.S., Tikoo S., Jackett L.A., Quek C., Brown D.L., Sharp D.M., Kwan R.Y.Q. (2019). RAB27A promotes melanoma cell invasion and metastasis via regulation of pro-invasive exosomes. Int. J. Cancer.

[23] Bahadoran P., Busca R., Chiaverini C., Westbroek W., Lambert J., Bille K., Valony G., Fukuda M., Naeyaert J.M., Ortonne J.P. (2003). Characterization of the molecular defects in Rab27a, caused by RAB27A missense mutations found in patients with Griscelli syndrome. J. Biol. Chem..

[24] Aslan D., Sari S., Derinöz O., Dalgiç B. (2006). Griscelli syndrome: Description of a case with Rab27A mutation. Pediatr. Hematol. Oncol..

[25] Bhattarai D., Banday A.Z., Joshi S., Adhikari R.C., Ali I., Rashid M., Kambay A.H. (2025). Griscelli Syndrome Type 2 Secondary to a Novel RAB27A Variant Presenting With Dermatitis. J. Cutan. Pathol..

[26] Maimaris J., Roa-Bautista A., Sohail M., Booth C., Cugno C., Chenchara L., Omran T.B., Hacohen Y., Lim M., Gilmour K. (2024). Griscelli Syndrome Type 2: Comprehensive Analysis of 149 New and Previously Described Patients with RAB27A Deficiency. J. Clin. Immunol..

[27] Rausch J., Herold S., Liebhäuser S., Bülbül Y., Antunes Ferreira E., Wenz T., Legscha K.J., Bros M., Butsch F., Kriege O. (2025). Case Report: Late-onset primary hemophagocytic lymphohistiocytosis leading to the diagnosis of Griscelli syndrome type 2 in a young woman with phenotypically inapparent partial albinism. Front. Immunol..

[28] Dornbos P., Singh P., Jang D.K., Mahajan A., Biddinger S.B., Rotter J.I., McCarthy M.I., Flannick J. (2022). Evaluating human genetic support for hypothesized metabolic disease genes. Cell Metab..

[29] Boucher J.M., Robich M., Scott S.S., Yang X., Ryzhova L., Turner J.E., Pinz I., Liaw L. (2018). Rab27a Regulates Human Perivascular Adipose Progenitor Cell Differentiation. Cardiovasc. Drugs Ther..

[30] Xiao Q., Zhao X.Y., Jiang R.C., Chen X.H., Zhu X., Chen K.F., Chen S.Y., Zhang X.L., Qin Y., Liu Y.H. (2019). Increased expression of Sonic hedgehog restores diabetic endothelial progenitor cells and improves cardiac repair after acute myocardial infarction in diabetic mice. Int. J. Mol. Med..

[31] Hu M., Guo G., Huang Q., Cheng C., Xu R., Li A., Liu N., Liu S. (2018). The harsh microenvironment in infarcted heart accelerates transplanted bone marrow mesenchymal stem cells injury: The role of injured cardiomyocytes-derived exosomes. Cell Death Dis..

[32] Kasai K., Ohara-Imaizumi M., Takahashi N., Mizutani S., Zhao S., Kikuta T., Kasai H., Nagamatsu S., Gomi H., Izumi T. (2005). Rab27a mediates the tight docking of insulin granules onto the plasma membrane during glucose stimulation. J. Clin. Investig..

[33] Yamaoka M., Ishizaki T., Kimura T. (2015). Interplay between Rab27a effectors in pancreatic β-cells. World J. Diabetes.

[34] Sun X., Lin J., Zhang Y., Kang S., Belkin N., Wara A.K., Icli B., Hamburg N.M., Li D., Feinberg M.W. (2016). MicroRNA-181b Improves Glucose Homeostasis and Insulin Sensitivity by Regulating Endothelial Function in White Adipose Tissue. Circ. Res..

[35] Liu Y., Sun Y., Lin X., Zhang D., Hu C., Liu J., Zhu Y., Gao A., Han H., Chai M. (2022). Perivascular adipose-derived exosomes reduce macrophage foam cell formation through miR-382-5p and the BMP4-PPARγ-ABCA1/ABCG1 pathways. Vasc. Pharmacol..

[36] Wei X., Guan X., Ma X., He X., Wang S., Zhou G., Liu H., Fan Y. (2025). Neutrophil membrane-engineered extrcellular vesicles from perivascular adipose tissue-derived stromal cells for atherosclerotic lesion resolution and enhanced vascular grant remodeling. Adv. Funct. Mater..

[37] Xie X., Wang S., Rao J., Xue J., Lin K., Lin N., Li K., Wu S., Liang W., Guo Y. (2022). Comprehensive Analysis of Differentially Expressed lncRNAs in the Perivascular Adipose Tissue of Patients with Coronary Heart Disease. Rev. Cardiovasc. Med..

[38] Ghadami S., Dellinger K. (2023). The lipid composition of extracellular vesicles: Applications in diagnostics and therapeutic delivery. Front. Mol. Biosci..

[39] Durcin M., Fleury A., Taillebois E., Hilairet G., Krupova Z., Henry C., Truchet S., Trotzmuller M., Kofeler H., Mabilleau G. (2017). Characterisation of adipocyte-derived extracellular vesicle subtypes identifies distinct protein and lipid signatures for large and small extracellular vesicles. J. Extracell. Vesicles.

[40] Khanabdali R., Shojaee M., Johnson J., Law S.Q.K., Lim M.B.L., James P.F., Tester A., Kalionis B. (2025). Profiling the extracellular vesicles of two human placenta-derived mesenchymal stromal cell populations. Exp. Cell Res..

[41] Gummadi S., Chitti S.V., Kang T., Shahi S., Mathivanan S., Fonseka P. (2025). ExoCarta 2024: A Web-based Repository of Small Extracellular Vesicles Cargo. J. Mol. Biol..

[42] Anwar M.Y., Highland H.M., Palmer A.B., Duong T., Lin Z., Zhu W., Sprinkles J., Kim D., Young K.L., Chen H.H. (2025). The Circulating Lipidome In Severe Obesity. medRxiv.

[43] Pizzinat N., Ong-Meang V., Bourgailh-Tortosa F., Blanzat M., Perquis L., Cussac D., Parini A., Poinsot V. (2020). Extracellular vesicles of MSCs and cardiomyoblasts are vehicles for lipid mediators. Biochimie.

[44] Watanabe Y., Tatsuno I. (2020). Prevention of Cardiovascular Events with Omega-3 Polyunsaturated Fatty Acids and the Mechanism Involved. J. Atheroscler. Thromb..

[45] Cartier A., Hla T. (2019). Sphingosine 1-phosphate: Lipid signaling in pathology and therapy. Science.

[46] Cartier A., Leigh T., Liu C.H., Hla T. (2020). Endothelial sphingosine 1-phosphate receptors promote vascular normalization and antitumor therapy. Proc. Natl. Acad. Sci. USA.

[47] Hilvo M., Vasile V.C., Donato L.J., Hurme R., Laaksonen R. (2020). Ceramides and Ceramide Scores: Clinical Applications for Cardiometabolic Risk Stratification. Front. Endocrinol..

[48] Kopprasch S., Dheban S., Schuhmann K., Xu A., Schulte K.M., Simeonovic C.J., Schwarz P.E., Bornstein S.R., Shevchenko A., Graessler J. (2016). Detection of Independent Associations of Plasma Lipidomic Parameters with Insulin Sensitivity Indices Using Data Mining Methodology. PLoS ONE.

[49] Bhat O.M., Mir R.A., Nehvi I.B., Wani N.A., Dar A.H., Zargar M.A. (2024). Emerging role of sphingolipids and extracellular vesicles in development and therapeutics of cardiovascular diseases. Int. J. Cardiol. Heart Vasc..

[50] Jiang X.C., Liu J. (2013). Sphingolipid Metabolism and Atherosclerosis. Sphingolipids in Disease.

[51] Donoso-Quezada J., Ayala-Mar S., González-Valdez J. (2021). The role of lipids in exosome biology and intercellular communication: Function, analytics and applications. Traffic.

[52] Bieberich E. (2018). Sphingolipids and lipid rafts: Novel concepts and methods of analysis. Chem. Phys. Lipids.

[53] Verderio C., Gabrielli M., Giussani P. (2018). Role of sphingolipids in the biogenesis and biological activity of extracellular vesicles. J. Lipid Res..

[54] Mahmoud A.M., Mirza I., Metwally E., Morsy M.H., Scichilone G., Asada M.C., Mostafa A., Bianco F.M., Ali M.M., Masrur M.A. (2025). Lipidomic profiling of human adiposomes identifies specific lipid shifts linked to obesity and cardiometabolic risk. JCI Insight.

[55] Quinville B.M., Deschenes N.M., Ryckman A.E., Walia J.S. (2021). A Comprehensive Review: Sphingolipid Metabolism and Implications of Disruption in Sphingolipid Homeostasis. Int. J. Mol. Sci..

[56] Yaribeygi H., Bo S., Ruscica M., Sahebkar A. (2020). Ceramides and diabetes mellitus: An update on the potential molecular relationships. Diabet. Med..

[57] Zhu Q., Scherer P.E. (2024). Ceramides and Atherosclerotic Cardiovascular Disease: A Current Perspective. Circulation.

[58] Choi R.H., Tatum S.M., Symons J.D., Summers S.A., Holland W.L. (2021). Ceramides and other sphingolipids as drivers of cardiovascular disease. Nat. Rev. Cardiol..

[59] Vessey D.A., Li L., Kelley M., Zhang J., Karliner J.S. (2008). Sphingosine can pre- and post-condition heart and utilizes a different mechanism from sphingosine 1-phosphate. J. Biochem. Mol. Toxicol..

[60] Vessey D.A., Li L., Honbo N., Karliner J.S. (2009). Sphingosine 1-phosphate is an important endogenous cardioprotectant released by ischemic pre- and postconditioning. Am. J. Physiol.-Heart Circ. Physiol..

[61] Egom E.E., Mohamed T.M., Mamas M.A., Shi Y., Liu W., Chirico D., Stringer S.E., Ke Y., Shaheen M., Wang T. (2011). Activation of Pak1/Akt/eNOS signaling following sphingosine-1-phosphate release as part of a mechanism protecting cardiomyocytes against ischemic cell injury. Am. J. Physiol.-Heart Circ. Physiol..

[62] Egea-Jimenez A.L., Zimmermann P. (2020). Lipids in Exosome Biology. Handb. Exp. Pharmacol..

[63] Subra C., Grand D., Laulagnier K., Stella A., Lambeau G., Paillasse M., De Medina P., Monsarrat B., Perret B., Silvente-Poirot S. (2010). Exosomes account for vesicle-mediated transcellular transport of activatable phospholipases and prostaglandins. J. Lipid Res..

[64] Molina Y.L., García-Seisdedos D., Babiy B., Lerma M., Martínez-Botas J., Casarejos M.J., Vallejo M.T., Gómez-Coronado D., Lasunción M.A., Pastor Ó. (2022). Rottlerin Stimulates Exosome/Microvesicle Release Via the Increase of Ceramide Levels Mediated by Ampk in an In Vitro Model of Intracellular Lipid Accumulation. Biomedicines.

[65] Elmallah M.I.Y., Ortega-Deballon P., Hermite L., Pais-De-Barros J.P., Gobbo J., Garrido C. (2022). Lipidomic profiling of exosomes from colorectal cancer cells and patients reveals potential biomarkers. Mol. Oncol..

[66] Brouwers J.F., Aalberts M., Jansen J.W., van Niel G., Wauben M.H., Stout T.A., Helms J.B., Stoorvogel W. (2013). Distinct lipid compositions of two types of human prostasomes. Proteomics.

[67] Samouillan V., Martinez de Lejarza Samper I.M., Amaro A.B., Vilades D., Dandurand J., Casas J., Jorge E., de Gonzalo Calvo D., Gallardo A., Lerma E. (2020). Biophysical and Lipidomic Biomarkers of Cardiac Remodeling Post-Myocardial Infarction in Humans. Biomolecules.

[68] Lee Y.C., Chang H.H., Chiang C.L., Liu C.H., Yeh J.I., Chen M.F., Chen P.Y., Kuo J.S., Lee T.J. (2011). Role of perivascular adipose tissue-derived methyl palmitate in vascular tone regulation and pathogenesis of hypertension. Circulation.

[69] Omachi D.O., Aryee A.N.A., Onuh J.O. (2024). Functional Lipids and Cardiovascular Disease Reduction: A Concise Review. Nutrients.

[70] Endo J., Arita M. (2016). Cardioprotective mechanism of omega-3 polyunsaturated fatty acids. J. Cardiol..

[71] Su H., Rustam Y.H., Masters C.L., Makalic E., McLean C.A., Hill A.F., Barnham K.J., Reid G.E., Vella L.J. (2021). Characterization of brain-derived extracellular vesicle lipids in Alzheimer’s disease. J. Extracell. Vesicles.

[72] Simbari F., McCaskill J., Coakley G., Millar M., Maizels R.M., Fabriás G., Casas J., Buck A.H. (2016). Plasmalogen enrichment in exosomes secreted by a nematode parasite versus those derived from its mouse host: Implications for exosome stability and biology. J. Extracell. Vesicles.

[73] Lydic T.A., Townsend S., Adda C.G., Collins C., Mathivanan S., Reid G.E. (2015). Rapid and comprehensive ‘shotgun’ lipidome profiling of colorectal cancer cell derived exosomes. Methods.

[74] Almsherqi Z.A. (2021). Potential Role of Plasmalogens in the Modulation of Biomembrane Morphology. Front. Cell Dev. Biol..

[75] Messias M.C.F., Mecatti G.C., Priolli D.G., de Oliveira Carvalho P. (2018). Plasmalogen lipids: Functional mechanism and their involvement in gastrointestinal cancer. Lipids Health Dis..

[76] Astudillo A.M., Balboa M.A., Balsinde J. (2023). Compartmentalized regulation of lipid signaling in oxidative stress and inflammation: Plasmalogens, oxidized lipids and ferroptosis as new paradigms of bioactive lipid research. Prog. Lipid Res..

[77] Chatterjee T.K., Stoll L.L., Denning G.M., Harrelson A., Blomkalns A.L., Idelman G., Rothenberg F.G., Neltner B., Romig-Martin S.A., Dickson E.W. (2009). Proinflammatory phenotype of perivascular adipocytes: Influence of high-fat feeding. Circ. Res..

[78] Takaoka M., Nagata D., Kihara S., Shimomura I., Kimura Y., Tabata Y., Saito Y., Nagai R., Sata M. (2009). Periadventitial adipose tissue plays a critical role in vascular remodeling. Circ. Res..

[79] Marchesi C., Ebrahimian T., Angulo O., Paradis P., Schiffrin E.L. (2009). Endothelial nitric oxide synthase uncoupling and perivascular adipose oxidative stress and inflammation contribute to vascular dysfunction in a rodent model of metabolic syndrome. Hypertension.

[80] Karastergiou K., Evans I., Ogston N., Miheisi N., Nair D., Kaski J.C., Jahangiri M., Mohamed-Ali V. (2010). Epicardial adipokines in obesity and coronary artery disease induce atherogenic changes in monocytes and endothelial cells. Arterioscler. Thromb. Vasc. Biol..

[81] Virdis A., Duranti E., Rossi C., Dell’Agnello U., Santini E., Anselmino M., Chiarugi M., Taddei S., Solini A. (2015). Tumour necrosis factor-alpha participates on the endothelin-1/nitric oxide imbalance in small arteries from obese patients: Role of perivascular adipose tissue. Eur. Heart J..

[82] Cheng K.H., Chu C.S., Lee K.T., Lin T.H., Hsieh C.C., Chiu C.C., Voon W.C., Sheu S.H., Lai W.T. (2008). Adipocytokines and proinflammatory mediators from abdominal and epicardial adipose tissue in patients with coronary artery disease. Int. J. Obes..

[83] Weston A.H., Egner I., Dong Y., Porter E.L., Heagerty A.M., Edwards G. (2013). Stimulated release of a hyperpolarizing factor (ADHF) from mesenteric artery perivascular adipose tissue: Involvement of myocyte BKCa channels and adiponectin. Br. J. Pharmacol..

[84] Irie D., Kawahito H., Wakana N., Kato T., Kishida S., Kikai M., Ogata T., Ikeda K., Ueyama T., Matoba S. (2015). Transplantation of periaortic adipose tissue from angiotensin receptor blocker-treated mice markedly ameliorates atherosclerosis development in apoE-/- mice. J. Renin Angiotensin Aldosterone Syst..

[85] Park K., Li Q., Lynes M.D., Yokomizo H., Maddaloni E., Shinjo T., St-Louis R., Li Q., Katagiri S., Fu J. (2022). Endothelial Cells Induced Progenitors Into Brown Fat to Reduce Atherosclerosis. Circ. Res..

[86] Qi X.Y., Qu S.L., Xiong W.H., Rom O., Chang L., Jiang Z.S. (2018). Perivascular adipose tissue (PVAT) in atherosclerosis: A double-edged sword. Cardiovasc. Diabetol..

[87] Xia N., Horke S., Habermeier A., Closs E.I., Reifenberg G., Gericke A., Mikhed Y., Münzel T., Daiber A., Förstermann U. (2016). Uncoupling of Endothelial Nitric Oxide Synthase in Perivascular Adipose Tissue of Diet-Induced Obese Mice. Arterioscler. Thromb. Vasc. Biol..

[88] Leandro A., Queiroz M., Azul L., Seiça R., Sena C.M. (2021). Omentin: A novel therapeutic approach for the treatment of endothelial dysfunction in type 2 diabetes. Free. Radic. Biol. Med..

[89] Chen Y., Liu F., Han F., Lv L., Tang C.E., Xie Z., Luo F. (2020). Omentin-1 is associated with atrial fibrillation in patients with cardiac valve disease. BMC Cardiovasc. Disord..

[90] Du Y., Ji Q., Cai L., Huang F., Lai Y., Liu Y., Yu J., Han B., Zhu E., Zhang J. (2016). Association between omentin-1 expression in human epicardial adipose tissue and coronary atherosclerosis. Cardiovasc. Diabetol..

[91] Harada K., Shibata R., Ouchi N., Tokuda Y., Funakubo H., Suzuki M., Kataoka T., Nagao T., Okumura S., Shinoda N. (2016). Increased expression of the adipocytokine omentin in the epicardial adipose tissue of coronary artery disease patients. Atherosclerosis.

[92] Mestres-Arenas A., Villarroya J., Giralt M., Villarroya F., Peyrou M. (2021). A Differential Pattern of Batokine Expression in Perivascular Adipose Tissue Depots from Mice. Front. Physiol..

[93] Chang L., Villacorta L., Li R., Hamblin M., Xu W., Dou C., Zhang J., Wu J., Zeng R., Chen Y.E. (2012). Loss of perivascular adipose tissue on peroxisome proliferator-activated receptor-γ deletion in smooth muscle cells impairs intravascular thermoregulation and enhances atherosclerosis. Circulation.

[94] Liu F., Fang S., Liu X., Li J., Wang X., Cui J., Chen T., Li Z., Yang F., Tian J. (2020). Omentin-1 protects against high glucose-induced endothelial dysfunction via the AMPK/PPARδ signaling pathway. Biochem. Pharmacol..

[95] Watts S.W., Shaw S., Burnett R., Dorrance A.M. (2011). Indoleamine 2,3-diooxygenase in periaortic fat: Mechanisms of inhibition of contraction. Am. J. Physiol.-Heart Circ. Physiol..

[96] Jüttner A.A., Ataei Ataabadi E., Golshiri K., de Vries R., Garrelds I.M., Danser A.H.J., Visser J.A., Roks A.J.M. (2024). Adiponectin secretion by perivascular adipose tissue supports impaired vasodilation in a mouse model of accelerated vascular smooth muscle cell and adipose tissue aging. Vasc. Pharmacol..

[97] Saxton S.N., Ryding K.E., Aldous R.G., Withers S.B., Ohanian J., Heagerty A.M. (2018). Role of Sympathetic Nerves and Adipocyte Catecholamine Uptake in the Vasorelaxant Function of Perivascular Adipose Tissue. Arterioscler. Thromb. Vasc. Biol..

[98] Greenstein A.S., Khavandi K., Withers S.B., Sonoyama K., Clancy O., Jeziorska M., Laing I., Yates A.P., Pemberton P.W., Malik R.A. (2009). Local inflammation and hypoxia abolish the protective anticontractile properties of perivascular fat in obese patients. Circulation.

[99] Agabiti-Rosei C., Saxton S.N., De Ciuceis C., Lorenza Muiesan M., Rizzoni D., Agabiti Rosei E., Heagerty A.M. (2024). Influence of Perivascular Adipose Tissue on Microcirculation: A Link Between Hypertension and Obesity. Hypertension.

[100] Okamoto Y., Kihara S., Ouchi N., Nishida M., Arita Y., Kumada M., Ohashi K., Sakai N., Shimomura I., Kobayashi H. (2002). Adiponectin reduces atherosclerosis in apolipoprotein E-deficient mice. Circulation.

[101] Astapova O., Leff T. (2012). Adiponectin and PPARγ: Cooperative and interdependent actions of two key regulators of metabolism. Vitam. Horm..

[102] Ayers S.D., Nedrow K.L., Gillilan R.E., Noy N. (2007). Continuous nucleocytoplasmic shuttling underlies transcriptional activation of PPARgamma by FABP4. Biochemistry.

[103] Margaritis M., Antonopoulos A.S., Digby J., Lee R., Reilly S., Coutinho P., Shirodaria C., Sayeed R., Petrou M., De Silva R. (2013). Interactions between vascular wall and perivascular adipose tissue reveal novel roles for adiponectin in the regulation of endothelial nitric oxide synthase function in human vessels. Circulation.

[104] Greif M., Becker A., von Ziegler F., Lebherz C., Lehrke M., Broedl U.C., Tittus J., Parhofer K., Becker C., Reiser M. (2009). Pericardial adipose tissue determined by dual source CT is a risk factor for coronary atherosclerosis. Arterioscler. Thromb. Vasc. Biol..

[105] Schroeter M.R., Eschholz N., Herzberg S., Jerchel I., Leifheit-Nestler M., Czepluch F.S., Chalikias G., Konstantinides S., Schäfer K. (2013). Leptin-dependent and leptin-independent paracrine effects of perivascular adipose tissue on neointima formation. Arterioscler. Thromb. Vasc. Biol..

[106] Gil-Ortega M., Condezo-Hoyos L., García-Prieto C.F., Arribas S.M., González M.C., Aranguez I., Ruiz-Gayo M., Somoza B., Fernández-Alfonso M.S. (2014). Imbalance between pro and anti-oxidant mechanisms in perivascular adipose tissue aggravates long-term high-fat diet-derived endothelial dysfunction. PLoS ONE.

[107] Hiramatsu-Ito M., Shibata R., Ohashi K., Uemura Y., Kanemura N., Kambara T., Enomoto T., Yuasa D., Matsuo K., Ito M. (2016). Omentin attenuates atherosclerotic lesion formation in apolipoprotein E-deficient mice. Cardiovasc. Res..

[108] Wang W.F., Li S.M., Ren G.P., Zheng W., Lu Y.J., Yu Y.H., Xu W.J., Li T.H., Zhou L.H., Liu Y. (2015). Recombinant murine fibroblast growth factor 21 ameliorates obesity-related inflammation in monosodium glutamate-induced obesity rats. Endocrine.

[109] Zeng Z., Zheng Q., Chen J., Tan X., Li Q., Ding L., Zhang R., Lin X. (2020). FGF21 mitigates atherosclerosis via inhibition of NLRP3 inflammasome-mediated vascular endothelial cells pyroptosis. Exp. Cell Res..

[110] Jin L., Lin Z., Xu A. (2016). Fibroblast Growth Factor 21 Protects against Atherosclerosis via Fine-Tuning the Multiorgan Crosstalk. Diabetes Metab. J..

[111] Zhang J., Cheng Y., Gu J., Wang S., Zhou S., Wang Y., Tan Y., Feng W., Fu Y., Mellen N. (2016). Fenofibrate increases cardiac autophagy via FGF21/SIRT1 and prevents fibrosis and inflammation in the hearts of Type 1 diabetic mice. Clin. Sci..

[112] Wu L., Qian L., Zhang L., Zhang J., Zhou J., Li Y., Hou X., Fang Q., Li H., Jia W. (2020). Fibroblast Growth Factor 21 is Related to Atherosclerosis Independent of Nonalcoholic Fatty Liver Disease and Predicts Atherosclerotic Cardiovascular Events. J. Am. Heart Assoc..

[113] Emont M.P., Jacobs C., Essene A.L., Pant D., Tenen D., Colleluori G., Di Vincenzo A., Jørgensen A.M., Dashti H., Stefek A. (2022). A single-cell atlas of human and mouse white adipose tissue. Nature.

[114] Sun W., Dong H., Balaz M., Slyper M., Drokhlyansky E., Colleluori G., Giordano A., Kovanicova Z., Stefanicka P., Balazova L. (2020). snRNA-seq reveals a subpopulation of adipocytes that regulates thermogenesis. Nature.

[115] Angueira A.R., Sakers A.P., Holman C.D., Cheng L., Arbocco M.N., Shamsi F., Lynes M.D., Shrestha R., Okada C., Batmanov K. (2021). Defining the lineage of thermogenic perivascular adipose tissue. Nat. Metab..

[116] Potts C.M., Yang X., Lynes M.D., Malka K., Liaw L. (2025). Exploration of Conserved Human Adipose Subpopulations Using Targeted Single-Nuclei RNA Sequencing Data Sets. J. Am. Heart Assoc..

[117] Vargas D., López C., Acero E., Benitez E., Wintaco A., Camacho J., Carreño M., Umaña J., Jimenez D., Díaz S. (2018). Thermogenic capacity of human periaortic adipose tissue is transformed by body weight. PLoS ONE.

[118] Vargas D., Camacho J., Duque J., Carreño M., Acero E., Pérez M., Ramirez S., Umaña J., Obando C., Guerrero A. (2017). Functional Characterization of Preadipocytes Derived from Human Periaortic Adipose Tissue. Int. J. Endocrinol..

[119] Doyle L.M., Wang M.Z. (2019). Overview of Extracellular Vesicles, Their Origin, Composition, Purpose, and Methods for Exosome Isolation and Analysis. Cells.

[120] Welsh J.A., Goberdhan D.C.I., O’Driscoll L., Buzas E.I., Blenkiron C., Bussolati B., Cai H., Di Vizio D., Driedonks T.A.P., Erdbrügger U. (2024). Minimal information for studies of extracellular vesicles (MISEV2023): From basic to advanced approaches. J. Extracell. Vesicles.

[121] Lee J.E., Moon P.G., Lee I.K., Baek M.C. (2015). Proteomic Analysis of Extracellular Vesicles Released by Adipocytes of Otsuka Long-Evans Tokushima Fatty (OLETF) Rats. Protein J..

[122] Xu X., Yin F., Guo M., Gan G., Lin G., Wen C., Wang J., Song P., Wang J., Qi Z.Q. (2023). Quantitative proteomic analysis of exosomes from umbilical cord mesenchymal stem cells and rat bone marrow stem cells. Proteomics.

[123] Men Y., Yelick J., Jin S., Tian Y., Chiang M.S.R., Higashimori H., Brown E., Jarvis R., Yang Y. (2019). Exosome reporter mice reveal the involvement of exosomes in mediating neuron to astroglia communication in the CNS. Nat. Commun..

[124] Fordjour F.K., Abuelreich S., Hong X., Chatterjee E., Lallai V., Ng M., Saftics A., Deng F., Carnel-Amar N., Wakimoto H. (2023). Exomap1 mouse: A transgenic model for in vivo studies of exosome biology. bioRxiv.

[125] Neckles V.N., Morton M.C., Holmberg J.C., Sokolov A.M., Nottoli T., Liu D., Feliciano D.M. (2019). A transgenic inducible GFP extracellular-vesicle reporter (TIGER) mouse illuminates neonatal cortical astrocytes as a source of immunomodulatory extracellular vesicles. Sci. Rep..

[126] Zhang E., Liu Y., Han C., Fan C., Wang L., Chen W., Du Y., Han D., Arnone B., Xu S. (2021). Visualization and Identification of Bioorthogonally Labeled Exosome Proteins Following Systemic Administration in Mice. Front. Cell Dev. Biol..

[127] Cersosimo A., Longo Elia R., Condello F., Colombo F., Pierucci N., Arabia G., Matteucci A., Metra M., Adamo M., Vizzardi E. (2025). Cardiac rehabilitation in patients with atrial fibrillation. Minerva Cardiol. Angiol..

[128] Abelhad N.I., Kachur S.M., Sanchez A., Lavie C.J., Milani R.V. (2023). Impact of cardiac rehabilitation on psychological factors, cardiorespiratory fitness, and survival: A narrative review. Heart Mind.

[129] Kim H., Jung J., Park S., Joo Y., Lee S., Sim J., Choi J., Lee H., Hwang G., Lee S. (2023). Exercise-Induced Fibroblast Growth Factor-21: A Systematic Review and Meta-Analysis. Int. J. Mol. Sci..

[130] Liu C., Yan X., Zong Y., He Y., Yang G., Xiao Y., Wang S. (2024). The effects of exercise on FGF21 in adults: A systematic review and meta-analysis. PeerJ.

[131] Jin L., Geng L., Ying L., Shu L., Ye K., Yang R., Liu Y., Wang Y., Cai Y., Jiang X. (2022). FGF21-Sirtuin 3 Axis Confers the Protective Effects of Exercise Against Diabetic Cardiomyopathy by Governing Mitochondrial Integrity. Circulation.

